# Address

**Published:** 1878-05

**Authors:** H. E. Beach


### ADDRESS.

*Before the Tennessee Dental Association at Lookout Mountain, June 1877.*

BY H. E. BEACH, D. D. S.

*Gentlemen of the Tennessee Dental Association:* Another year has elapsed since we met in council on the profession of our choice. We have had during that time, our successes and our failures.

Each of us I trust, has been profited by the experience of the past, and the counsel we received in our associated capacity

We have met to-day under the goodness and mercy of God, here on Lookout Mountain arrayed as it is in all the grandeur and beauty of nature's luxuriant wardrobe, to compare notes on our past experience and gather from them, that which is good, and tends to alleviate the sufferings of humanity.

I congratulate you on the good results already realized. From the experience of the past, we have much to encourage us, and yet there is much to lament.

With all our boasted scientific attainments, the best of us are too frequently made to mourn the loss of one or more of the priceless organs it is our privilige to preserve to its owner, but be it said to the honor of the profession, the blame oftener attaches to the owner than the operator.

Much has already been done and yet there is much to be done, to relieve unfortunate and suffering humanity.

The world with its countless millions is now looking to us for the preservation of the teeth, and the treatment of the diseases of the oral cavity. Our field of labor is a wide one, it is an honorable one. Our profession is distinct and should stand alone in its individuality.

What the royal road that leads to it is, is a question yet to be settled by those who ever strive to attain a higher degree of excellence; but as I have already said it is an honorable one, made so by men who first "made themselves honorable" and then by their association together, have made the profession what it is to-day.

Many important questions will here be brought before you for consideration, the discussion of which will, I trust be marked with that courtesy which has ever characterized your deliberations in the past.

As presiding officer it is my purpose to extend the broadest latitude consistent with the good order and decorum of the meeting, and if I err in the discharge of my duty I know you will extend to me that charity which should ever be accorded to one committing an error of the head and not of the heart.

Of the many topics for consideration that will be brought before you, I desire to call special attention to the following, viz: The causes and prevention ofdental caries, dental pathology and therapeutics, and the relation of dentist to his patient.

First. Recognizing the great fact that he who prevents the decay of a tooth, is deserving infinitely more credit, and a much higher rank in his profession, than he who plugs or extracts one, however skillfully either may be done, I would insist that you give the subject of caries and its prevention, your most careful consideration.

When the causes that act so destructively on the dental organ have been fully comprehended and mastered, then, and not until then, will you be able to prevent the ravages of caries. To the dentist whose science and skill teaches us to prevent the diseases we are called on to treat, will be entitled to the highest honors of the profession, as well as the everlasting gratitude and richest rewards of the world.

To be able to correct abnormal and destructive tendencies, which by their influence torture the unfortunate, and disgrace the oral cavity, will entitle you to the gratitude of your patrons as well as give you a peace and consolation that will whisper softly but sweetly to an easy conscience, "well done thou good and faithful servant." After all, it is not the pecuniary reward, but the intelligent exercise of skill, that is the sweet harbinger of peace to the scientific mind.

Second. Dental pathology and therapeutics demands more attention from the profession at large than it has heretofore received.

I am proud to believe that the working members of this association are fully alive to this important subject, but it is through your efforts that others are to be made to feel its importance, and bring themselves up to that standard of proficiency which will enable them to combat the diseases of the mouth incident to every practice.

From my observation, I am pursuaded that such is not now the case.

There are many who fancy themselves under the treatment of a skillful dentist, who find when it is too late they have suffered an irreparable loss from the pathological condition of the gums, and alveolar process; such should not be the case.

The last subject to which I invite your attention is the "relation of dentist to patient." I am not ignorant of the fact that this is clearly set forth in our code of professional ethics, but inasmuch as it has heretofore found but little space in your discussions, I am of opinion that much can be profitably learned by an occasional interchange of views in reference thereto. The dignity and purity of the profession, the high toned gentlemanly bearing of its members, its influence in society in educating the public to a proper understanding of the treatment necessary to the perfect development of the teeth of the young, to a great extent rests with our associations, as being made up of the representative members of the profession. Many of our patients are married ladies, from whom their offspring receive the aliments of nutrition, necessary to the development of the teeth. I think it is the province of the family dentist to advise the taking of such food as will most likely supply these aliments whenever it can be done without touching the sensibilities of the punctilious. The relation then becomes the most sacred between the family dentist and the patient of all others, save that of the family physician. No truly honorable gentleman should ever, in any way, refer to his patients by name in office conversation.

Facts may sometimes be mentioned by way of comparison or illustration, but always omit the name of the patient to whom you refer.

Holding to these views as sacred, I desire to have them impressed on the younger members of our profession, incorporated into their natures, and made a part of themselves.

